# A Simplified Method of Manually Constructing Small Format Tissue Microarray for Use in Resource-Constrained Settings

**DOI:** 10.30699/IJP.2023.562055.2972

**Published:** 2023-06-20

**Authors:** Seetu Palo

**Affiliations:** *Department* * of Pathology and Laboratory Medicine, All India Institute of Medical Sciences, Bibinagar, Telangana*

**Keywords:** Core flotation, Core tilt, Skin punch biopsy needles, Tissue microarray

## Abstract

**Background & Objective:**

Tissue microarray (TMA) is a method of harvesting small tissue cores from a number of donor paraffin tissue blocks and arraying them in a recipient paraffin block. It has numerous advantages and applications but is expensive. This study aimed to develop a simple yet efficient method of manual, small-format TMA block construction.

**Methods:**

Disposable skin punch biopsy needles were used to manually core out 4-mm cylinders from the archival donor blocks comprising tissue from 60 thyroidectomy specimens. These cores were oriented in the embedding cassette in accordance with the grid design. The molten wax was slowly dispensed and allowed to be set. Sectioning, mounting, and hematoxylin and eosin (H&E) staining were performed by a conventional method. Immunohistochemical studies, using HBME-1, CK19, and S100 antibodies, were also performed on these tissue array sections.

**Results:**

There was no core loss during processing. Technical issues like core tilt and floatation were easily tackled. Morphological identification, histological typing, and immunohistochemical analysis could be satisfactorily performed in these TMA sections. Donor blocks did not break after punching.

**Conclusion:**

This TMA construction method is simple, feasible, easily reproducible, and time-saving. It can serve as an excellent cost-effective alternative for resource-poor laboratories for carrying out immunohistochemical studies.

## Introduction

Tissue microarray (TMA) has revolutionized pathological research by paving the way for high-throughput molecular analysis in the field of diagnostic, prognostic, and predictive oncology. It is an innovative technique in which small tissue cores are extracted from a multitude of pre-existing paraffin-embedded donor tissue blocks and are precisely re-embedded onto a single recipient block at predefined array positions. In other words, TMAs are “micro-biopsies of biopsies” and can be potentially used for histochemical, immunohistochemical, immunofluorescence, and in situ hybridization studies (1,2). It allows simultaneous analysis of a large number of specimens and facilitates experimental uniformity while ensuring maximal preservation of archival tissue. However, one of the major disadvantages of current TMAs is their high cost, limiting their use in general practice (3). Commercially available TMA molds and automated tissue arrayers are expensive and clearly out of reach for many laboratories in the Indian subcontinent (4). Girish and Vijayalakshmi suggested that such institutes, which require TMAs, could send their blocks to apex institutes for construction (5). Other alternatives would be to devise a simple in-house TMA construction method that can be conveniently used when the need arises. Here, we present an easy and cost-effective manual technique for the construction of small-format TMAs using disposable skin punch biopsy needles.

## Material and Methods

**Fig. 1:**
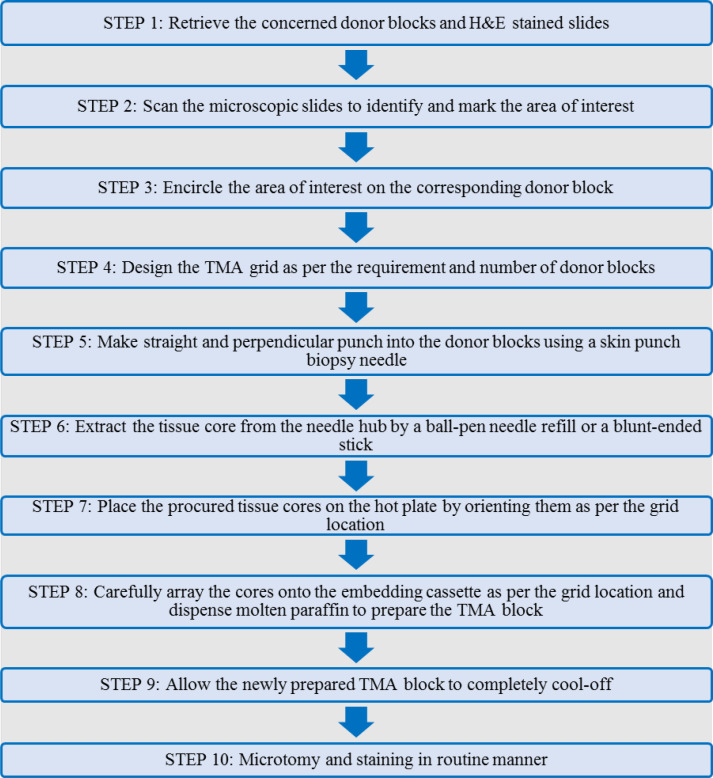
Schematic representation of the workflow of manual construction of small format tissue microarray (TMA)

**Fig. 2:**
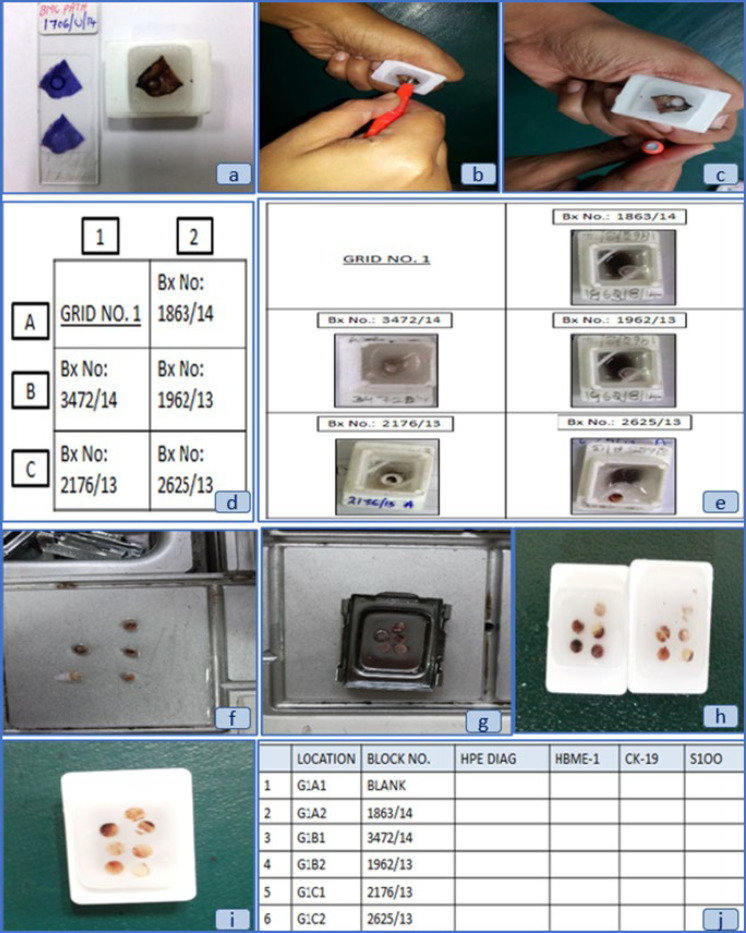
(a-j): Steps in constructing the tissue microarray TMA block - a) Marking the area of interest on the slide and then on the corresponding block; b & c) Punching and procuring tissue core with the help of skin punch biopsy needle; d) Layout of a 3x2 TMA grid diagram; e) Arranging the donor blocks according to the grid diagram; f) Placing the cores on hot plate in order to melt-off the paraffin; g) Arraying the cores onto the embedding cassette; h) 3x2 TMA blocks; i) 4x2 TMA block; j) Template of data entry sheet

A total of 15 TMA blocks were constructed manually using disposable skin core biopsy needles of a 4-mm bore diameter (without a plunger system). Tissues derived from 60 archival thyroidectomy specimens (10 hyperplastic nodules, 14 follicular adenomas, 16 classical papillary thyroid carcinomas, 15 follicular variant papillary thyroid carcinomas, 4 follicular carcinomas, and 1 medullary carcinoma) were used for array construction. Formalin-fixed paraffin-embedded blocks and hematoxylin and eosin (H&E) stained slides were retrieved and analyzed to define the region of interest. The representative areas on these H&E-stained slides were then demarcated using a felt-tip pen. The marked slides were overlaid on the corresponding paraffin blocks, and the same area was circled on the block using a permanent marker pen. Clean punches were made with the help of a skin core biopsy needle from the circled area of the donor paraffin block. Tissue cores were gently expressed out of the needle using the blunt end of the ball-pen refill. Thus, a core of tissue measuring 4 mm in diameter was procured from each block. TA total of 110 tissue cores (2 representative cores from the neoplastic cases and one representative core each from the 10 hyperplastic nodules) were taken in the study for constructing 15 TMA blocks of varying capacity. Before proceeding further, 3×2, 4×2, 3×3, and 4×3 grid diagrams were designed, indicating the position and biopsy number of each core. An empty space was left at the left-upper coordinate of each TMA design to serve as an orientation point during the microscopic assessment. These diagrams were used as a guide during arraying and microscopy. The obtained tissue cores were transferred onto a hot plate to completely melt the adhered paraffin. These cores were then arrayed onto the embedding cassette in accordance with the grid configuration. Melted paraffin was slowly dispensed onto the cassette without disturbing the cores’ positions. After all the TMA blocks were constructed, 3- to 5-μm thick sections were cut and stained with H&E. Immunohistochemical staining, using HBME-1, CK19, and S100 antibodies, was also performed according to the standard operating protocol. The step-by-step construction procedure is illustrated in Figures 1 and 2. 

## Results

Using the above method, 15 TMA blocks were constructed, comprising 3×2 (n=5), 4×2 (n=4), 3×3 (n=3), and 4×3 (n=3) grid designs. Once the lesional area was marked on the donor block, it took less than 7 min to construct a single TMA block (from punching to embedding). The overall construction procedure was quite easy and feasible. Minor technical difficulties (such as core tilt and floatation) at the time of pouring molten paraffin were easily sorted out by re-orienting and re-positioning the cores with blunt forceps. These difficulties were not encountered if the paraffin was dispensed little by little in small amounts and controlled manner. Regardless of the number of tissue cores, sections of 3- to 5-µm thickness could be easily cut, and none of the cores fell off the block during microtomy. The time required for cutting the sections did not differ between 2×3, 2×4, 3×3, and 3×4 array blocks. We did not observe any splitting of sections on the hot water bath or during mounting. Morphological identification, histological typing, and immunohistochemical analysis were easily and successfully done on TMA sections. None of the immunohistochemical slides had a core loss during antigen retrieval or washes. Also, there was no substantial damage to the donor blocks from the punches, and they retained their integrity and diagnostic utility.

## Discussion

In 1986, Battifora conceptualized and constructed a multi-tumor (sausage) tissue block to test new antibodies (6). Since then, many researchers have made utmost efforts to design, refine, and improve different TMA construction techniques using different punching tools and embedding mediums (7). Shebl *et al.* and Foda *et al.* constructed TMA blocks using a mechanical pencil tip, while Shi *et al.* used a blade-shaped knife self-made from a disposable microtome knife as their sampling tool (8,9,10). Pires and colleagues modified conventional hypodermic needles to punch tissue cores from donor blocks (11). Others have effectively used bone marrow trephine needle biopsies and skin punch biopsy needles (12,13,14,15). Santos *et al.* observed that TMA recipient blocks prepared from paraffin enriched with polymers produced slides with superior features compared to pure conventional paraffin and a mixture of pure conventional paraffin with 5%, 10%, or 15% of beeswax (16). Another modification was proposed by Yan and colleagues (17). They opined TMA blocks prepared using agarose-paraffin ensured limited tissue core loss during cutting and mounting and immunohistochemical or fluorescent *in situ* hybridization staining. Modern-day TMA technology has so remarkably evolved that even ultrahigh-density TMAs containing 10,000 samples per block are possible (18).

Besides excision biopsies, needle biopsies are also amenable to tissue sampling for TMA construction. Fridman *et al.* and McCarthy *et al.* constructed TMAs from prostate needle biopsies (19,20). Obermann *et al.* used the “stack method” to construct TMAs from bone marrow biopsies and validated the approach by comparing immunohistochemical results obtained from whole tissue sections and TMA sections (21). They also suggested that the stack method may be used for arraying other small tissue samples, such as renal or liver biopsies. TMAs can also be prepared from frozen tissues and cell lines (22).

The method described herein is a simple and inexpensive manual small-format TMA technique using the already existing infrastructure and consumables. The only thing that needs to be additionally procured is skin punch biopsy needles. These needles are cheap and can be reused multiple times. The below-listed care and precautions should be taken during construction to ensure a satisfactory end product.

1. The donor paraffin block should be pre-evaluated to roughly determine the depth of the tissue, which should be ideally more than 2 mm. The thinness of donor tissue will mean a lesser number of TMA slides and core loss.
2. While encircling the area on the block, do not press into the paraffin, or else the tip of the marker may get blocked with paraffin and might need frequent cleaning with a paper towel.
3. One should be cautious while handling the biopsy needle since it has a very sharp tip.
4. Prior to punching, arrange the blocks in order according to the grid design and keep referring to the grid coordinates at all the steps.
5. During punching, special care has to be taken to insert the needle perpendicularly into the block so that the cores extracted will have a perfect cylindrical shape.
6. Always follow the same pattern for constructing all TMA blocks. Start from the top-left coordinate and proceed from left to right and top to bottom.
7. Dispense the paraffin slowly and allow it to set in between to avoid core tilt and floatation.
8. Allow the freshly prepared TMA block to cool off completely before sectioning. This would ensure adequate annealing between the tissue core and paraffin so that the cores will not fall off the block.

In the conventional manual TMA construction procedure, a recipient TMA block with holes is first created, and then the tissue cylinders from the donor blocks are annealed within it. Srinath *et al.* fabricated a silicone-based master TMA mold that prepared TMA recipient blocks quite easily (4). Liquid wax was poured into this mold, and after solidification, the “holed” recipient block was ready (4). In our procedure, we directly hand-positioned the donor cores onto the embedding cassette, thereby significantly reducing the production time and labor. However, as a result of not using a prefabricated recipient block, core tilt and flotation were encountered during the embedding process, easily tackled by dispensing the molten paraffin in a controlled manner. Alternatively, to avoid these difficulties, different methods can be used during the embedding process, such as double-sided adhesive tape, encasement in a drinking straw, and paper molds (23,24,25-27). Such additional steps demand specific experience and technical skills that are time-consuming. In our method, once the paraffin of the TMA block sets, the blocks can be directly subjected to microtomy without the need for an additional melting process to establish strong contact between the paraffin and the tissue cores placed (12,25,28). On the flip side, our technique is only feasible when the number of cores is limited and the core diameter is more. It is observed that TMAs comprising less than 30 tissue cores per block can be comfortably constructed without the requirement of a prefabricated recipient block (26,29). By following our method and introducing customization, such as reducing the diameter of the tissue cores and the distance between them, a higher-density TMA block can be achieved. Komina and Petrusevska constructed 60 core-TMA using a skin punch biopsy needle of a 2-mm diameter (30). However, if multiple TMAs with a high number of specimens are to be constructed, then using a semi-automatic or automatic tissue arrayer may be favorable. 

The diameter of standard array needles varies from 0.6 to 2.0 mm, allowing about 300–500 cores and 50–100 cores per block, respectively (10,22,31). By minimizing the core diameter to 0.43 mm, a TMA block could accommodate up to 1,000 samples (32). Hence, smaller diameter cores allow for a greater number of cores to be placed in a single TMA block. On the other hand, larger-sized cores can be easily handled and arrayed. Additionally, they offer more tissue area for microscopic evaluation. Larger cores may be more beneficial when the investigators want to perform quantitative immunohistochemical analysis. According to Saxena and Badve, larger-sized cores increase the chances of donor blocks being broken or cracked during tissue extraction (33). This is in contrast with our observation, as none of the donor blocks were damaged after punching in our study sample. These donor blocks could be used for further conventional sectioning if required. In this study, it was found that 4-mm diameter tissue cores maintained recognizable histological features of the arrayed tissues and offered more area to quantitatively evaluate immunostaining patterns. We successfully performed immunodetection of HBME-1, CK-19, and S100 in the TMAs of thyroidectomy specimens. The staining results were in agreement with those of the whole slide section. The use of TMAs helped us to significantly cut down the usage of costly immunohistochemical consumables and the assay time.

## Conclusion

This manual TMA construction technique is simple, feasible, and cost-effective and requires less time and labor. The results of the immune-histochemical staining performed on TMAs are consistent with those performed on whole sections. Hence, both histomorphological and immune-histochemical analyses can be satisfactorily conducted on these blocks. Even though our TMA arrays contain a small number of donor samples, they are still a very good alternative for carrying out diagnostic and prognostic immunohistochemical research projects in many resource-poor laboratories. The described method is easily reproducible, and individual laboratories can modify the number of cores in the array and customize the grid design to fulfill their desired results and requirements.

## Conflict of Interest

The authors declared no conflict of interest.
